# A Case for Eustress in Grazing Animals

**DOI:** 10.3389/fvets.2019.00303

**Published:** 2019-09-13

**Authors:** Juan J. Villalba, Xavier Manteca

**Affiliations:** ^1^Department of Wildland Resources, Utah State University, Logan, UT, United States; ^2^School of Veterinary Science, Universidad Autónoma de Barcelona, Barcelona, Spain

**Keywords:** eustress, grazing, herbivore, animal health, adaptive behavior

## Abstract

Herbivores grazing in extensive systems are exposed to a series of challenges, rooted in the inherent spatial and temporal variability of their environment that potentially constrain their health, nutrition, and welfare. Nevertheless, in this review, we argue that challenges induced by some biotic (e.g., vegetation) and abiotic (e.g., terrain) factors may also be viewed as “positive” sources of stress or eustress, since they present complex problems, that when solved successfully elicit a greater degree of behavioral plasticity and adaptability in grazing animals. Chemically and structurally diverse landscapes require animals to display complex behaviors and exhibit adaptive capabilities, like building a balanced and safe diet or finding shelter, which ultimately lead to positive emotional states. Thus, maintaining or enhancing the diversity occurring in natural systems represent a management approach that can be used to improve welfare and prepare the animal for an efficient adaptation to future, and potentially unknown, environmental challenges.

## Introduction

Animal welfare is an essential element of modern animal production. First and foremost, animal welfare is grounded on ethical concerns that derive from the fact that animals are sentient beings, i.e., able to suffer and experience emotions, but improving animal welfare may have additional benefits. As many welfare problems have a detrimental effect on production, improving the welfare of farm animals very often has positive effects on performance (1, 2). In addition, improving animal welfare is one of the strategies that potentially contributes to reduce the use of antimicrobials in farm animals (3).

An in-depth discussion of the concept of animal welfare is well-beyond the scope of this review paper, and several reviews are available on the topic [e.g., (4–6)]. However, it is important to mention that animal welfare encompasses not only the physical health of the animals (i.e., the absence of diseases and injuries) but also their behavior and emotions (6–8).

For many years, the Five Freedoms (9) have provided a useful framework to identify the welfare problems of farm animals. These freedoms, which represent ideal states rather than actual standards for animal welfare are (a) freedom from thirst, hunger and malnutrition, (b) freedom form thermal and physical discomfort, (c) freedom from pain, injury and disease, (d) freedom to express most patterns of normal behavior, and (e) freedom from fear and distress.

More recently, the Five Freedoms have been criticized since they can be misunderstood as aiming at eliminating all negative experiences (which is not realistic or even desirable, as we argue in this review), but also because they fail to capture our current understanding of the biological processes underlying animal welfare (5). As an alternative to the Five Freedoms, the so-called Five Domains Model for assessing animal welfare was developed to address these problems. The Model incorporates four physical domains of “nutrition,” “environment,” “health,” and “behavior,” and a fifth “mental” domain. Each physical domain has an impact on the affective state of the animal (i.e., on the fifth domain), and the net outcome in the mental domain resulting from the combination of the four physical domains represents the animals' overall welfare state.

It is clear that the Five Domains Model applies to animals kept in confinement under intensive livestock production systems, but the approach is equally relevant to herbivores grazing in extensive systems, as all states identified in the model are crucial for the maintenance of their welfare, even when animals evolved and are presumably adapted to their “natural” grazing environments. In several instances, the natural environment negatively impacts animal welfare due to its inherent temporal and spatial variability, which may lead to unsuccessful coping responses to unpredictable and ever-changing challenges (10, 11). With regards to the grazing process, climate variability has significant negative effects on herbivores, particularly for dryland regions with low and variable precipitation and high temperatures in the growing season (12). Under these conditions, forage abundance and quality may be limited during certain periods, negatively affecting the nutrition domain, which in turn compromises animal welfare. Clear seasonal patterns have been observed for fecal cortisol levels (an indicator of chronic stress) in Pyrenean chamois (*Rupicapra pyrenaica*) grazing in rangelands of northeastern Spain (13). Concentration of fecal cortisol tracked the levels of nutrient and other environmental stresses experienced by these animals throughout the year (13). The effects of season are compounded with the impacts of thermal stress on ruminants in the face of future heat and cold waves of greater frequency, intensity and duration (14). As an example, heat stress reduces feed intake in ruminants while increasing maintenance requirements, a trend that is aggravated by the predicted decrease in the quality and productivity of feed resources available to herbivores grazing in rangelands (15). All these effects have the potential to promote reductions in animal welfare and productivity given that long-term declines in food availability lead to poor nutrition and stress (16). Another response related to the unpredictability observed in rangelands involves fear to predation and the indirect effects that predators impinge on prey by negatively affecting foraging patterns (i.e., reduced grazing time, increased vigilance), and as a consequence animal nutrition and reproduction rates (17, 18).

The aforementioned inherent variability of rangelands and the potential negative impacts on the nutrition and welfare of herbivores has been extensively reviewed elsewhere [e.g., (19) and papers in that book]. This review was developed with the aim of looking at variability in rangelands from a different angle, i.e., as a force that may potentially bring about greater adaptation and resiliency for animals grazing in the complex chemical and structural realm of rangelands. It is clear that welfare depends not only on whether the animal succeeds at coping with the challenges emerging from its environment, but also on whether coping attempts lead to negative consequences for the animal (20). On this regard, animals have evolved mechanisms to cope with different environmental challenges such that if they are to survive and reproduce, they should maintain a fitness level >0. Innate behavioral strategies and learning play a key role in the ability of animals to cope and adapt to stressful situations imposed by an ever-changing environment (21).

## Stress and Eustress in Extensive Systems

The concepts of “animal welfare” and “stress” are closely linked, partially because many welfare problems cause stress (22). The term “stress” has been widely used in biology to describe a set of physiological and behavioral changes elicited by aversive stimuli. Cannon (23) described stress as the sympatho-adrenomedullary (SAM) system's attempt to regulate homeostasis when threatened by a variety of aversive stimuli or stressors. Later, Selye (24) conducted some of his classic studies on the response of the hypothalamic-pituitary-adrenal (HPA) axis to noxious stimuli and suggested that the organism reacted in a non-specific manner to a wide variety of aversive stimuli, mainly with an increase in the HPA axis activity. Some forms of stress such as the chronic activation of the HPA axis caused by long-term factors are typically viewed as impinging deleterious effects on natural populations, inevitably resulting in maladaptation and pathology (25). Both the HPA axis and the SAM system are generally considered to be the two main elements of the stress response and levels of glucocorticoids in different animal tissues (e.g., blood, hair, saliva) and excretions (e.g., feces) have been widely used as measures of stress. The problem with this approach, however, is that the HPA axis and the SAM system have a crucial function in energy mobilization and redistribution of nutrients to active tissues and both aversive (e.g., fighting) and rewarding situations (e.g., play and mating) may elicit a similar physiological stress response (26). Therefore, if stress is perceived as potentially negative, it may be misleading to consider stress as a synonymous of the HPA axis activation. On the other hand, there is now enough evidence showing that it is not the physical nature of an aversive stimulus that has negative consequences on the animal but rather the degree to which the stimulus can be predicted and controlled (26). As a result, it has been suggested that the term “stress” should be restricted to conditions where an environmental demand exceeds the regulatory capacity of the organism, mainly when such conditions include unpredictability and uncontrollability (26). Thus, when animals have available the conditions or “tools” to control or predict their environment, a challenge may represent a stimulus that results in improvements to their welfare, provided that the animal overcomes the challenge. Interestingly, research in zoo animals has shown that giving animals the opportunity to choose between two different environments (which presumably increases the animals' perception of control) reduces several behavioral and physiological indicators of stress and poor welfare (27, 28).

The coping process to a stressful situation (i.e., an animal being exposed to a certain uncomfortable environment) may lead to fitness costs (i.e., searching activities that increase energy expenditure). Nevertheless, the net result of this response needs to be adaptive (i.e., finding a more comfortable environment) if the animal is to survive and reproduce, and if improvements in animal welfare are expected. Thus, under circumstances when the animal is able to fully cope to a challenge, this may have a positive impact on animal welfare. Under this context, the word “eustress” was coined (24) to refer to the idea that there is a “correct or optimal stress level” that is adaptive (29). For instance, problems that emerge during the grazing process such as building a nutritious and safe diet may be stressful (e.g., overcoming food neophobia, or preventing the ingestion toxic plants or excessive amounts of nutrients), like the example described above in zoo animals exposed to a choice between different environments, but ultimately beneficial if the individual possesses the skills and resources needed to meet such challenge. Problem-solving opportunities presented during enrichment programs for captive animals potentially enhances welfare, as individuals may be motivated to participate in problem-solving activities when there is an optimal level of challenge, which depends on the individual's cognitive and behavioral skills to solve the presented problem (30). Contrafreeloading, the choice to work for resources when identical resources are simultaneously available in free form, also entails an enrichment of the captive animal's environment as it provides opportunities for general exploration and cognitive challenge that may result in a positive outcome (i.e., “earning food”), even when such activities represent departures from optimal foraging strategies (31).

Our thesis in this review is that the inherent variability in structure, taxonomy, and chemistry provided by rangelands represent stimuli that enhance the adaptability and resiliency of herbivores grazing in these dynamic environments. This is a novel approach to managing animals in rangelands as it suggests that preserving and promoting rangeland diversity is crucial for “providing the training grounds” that will prepare animals to better respond and adapt to future challenges that compromise their welfare. We also submit that management interventions can contribute to foster flexibility in animals grazing in variable environments by providing the means and facilitating the acquisition of skills that optimize the prediction and control of the coping response to the problems presented in the context of a changing world.

## Forage Diversity and Eustress

It is known that a variety of plant species enhances the nutrition of mammalian herbivores because no single plant provides all the nutrients or proportions needed by the animal (32). In addition, plant secondary compounds (PSC) ingested as a dilute mixture of plants are less toxic to herbivores because they are less concentrated and potentially detoxified by different pathways [i.e., the Toxin Dilution Hypothesis; (33)]. Anatomical, physiological and experiential differences among individuals lead to specific needs, and thus individual animals can best meet their needs for nutrients and medicines when offered a multiplicity of forages, instead of receiving a single food, even if that food is balanced to meet the “average” needs of the “average” animal (34).

When herbivores engage in the process of building a diet from an array of different foods from a diverse plant community they are faced with solving a problem. This is because they need to balance the ingestion of required nutrients and potential medicines (35) from an array of nutritionally unbalanced and potentially toxic foods. The solution is achieved by the application of a suite of complex behaviors that require cognitive and non-cognitive mechanisms for their efficient execution in time and space (35, 36). For instance, locomotion activities position the individual in space, within the preferred patch and feeding station (36), followed by handling and ingestive activities that consummate a preference for particular plants and parts. Such preference is triggered by learning mechanisms that integrate the plant's orosensorial characteristics with its post-ingestive consequences (37, 38). The challenge of building a balanced diet from a diverse array of alternatives may “breed” innovation and exploratory behaviors in herbivores that when successful, foster positive emotional responses and allow for better adaptations to future unpredictable conditions of the environment (30). Good welfare is not simply the absence of negative experiences; positive affective states play a significant role in providing animals a better quality of life (39). Under this analysis, forage diversity could be interpreted as an “eustressor” that gives individuals the challenge but also the opportunity to execute behaviors efficiently across contexts and solve problems with potential to improve their welfare. In support of this idea, lambs faced with the problem of building a diet from a diverse array of food items with or without PSC, showed greater acceptance of novel foods and flavors in familiar (40) and novel (41) environments, and showed lower levels of stress-induced hyperthermia and ambulation scores in open field tests than animals exposed early in life to a single ration (41). Although the initial reaction to novel feeds in all treatments was similar (i.e., low food intake; neophobia), neophobia was attenuated at a quicker rate in animals that had the task of building a diet from single foods relative to those previously exposed to a single ration (40). In another study, lambs challenged to build a diet from an array of foods with different energy to protein ratios showed lower blood cortisol levels and neutrophil to lymphocyte ratios than lambs fed a single ration (42). Additionally, lambs under the diet-building task also spent a lower proportion of time eating and showed greater intake rates and greater proportion of time lying and greater activity than lambs under a single diet (42). Heifers grazing 2- or 3-way choices of different legumes showed greater body weight gains, forage intake (43) and hair cortisol levels than heifers grazing monocultures of these species. Some non-overwhelming challenges that increase cortisol levels, with the subsequent decline when the task is mastered or the stimulus removed, have been linked to the development of resilience and the fostering of adaptations that enhance emotional processing, cognitive control, and curiosity in monkeys (44).

Being able to solve a foraging problem, in addition to the reward provided by the nutrients harvested in the process (nutrition and behavior domains in the Five Domains Model), represents an intrinsic reward and positive emotional state (mental domain in the Five Domains Model) inherent to being successful at solving the task performed (30). Thus, the process of building a balanced and safe diet from a diverse array of nutritionally unbalanced alternatives may be interpreted as an achievement [a sense of “victory;” (24)] that leads to a positive emotional state that improves welfare. More research is clearly needed to better understand the effects of forage diversity and diet building on positive emotions in grazing animals. Nevertheless, since the display of behaviors concerning essential activities such as foraging are considered self-rewarding (39), it is plausible to speculate that successful diet building activities are also linked to positive emotional states. Food seeking (motivational states of wanting) and consummatory behaviors (liking or the hedonic pleasure felt during food consumption) have rewarding properties (45) and they are clearly involved in the process of diet selection in mammalian herbivores (35, 37). In addition, it has been shown that controlling an event *per se* can be perceived as rewarding or at least as less stressful (46). In contrast, foragers may experience negative states (i.e., frustration) when exposed to monotonous rations that may not satisfy all their individual and specific nutritional and medicinal requirements, as well as the need to experience a diverse array of flavors during the foraging process (47, 48). Under the context of single feeds or rations, foraging opportunities are limited since the only responses possible are to eat or to stop eating. Single foods/plants may elicit frustration as the animal's response does not lead to a solution, i.e., there are no single plants/forages that provide all the nutrients and proportions required by the animal (32, 34) and generalist herbivores evolved consuming a diversity of flavors from diverse plant communities instead of single flavors in monotonous foods. In addition, the initial stress (i.e., neophobia) promoted by exposing animals to diverse novel foods may be attenuated by the presence of a familiar model such as mother (35) or experienced companions (49), which allow for a prompt selection of a diversity of food items from the array of novel foods presented. Finally, forage diversity allows for the expression of foraging preferences that may not be able to be expressed under monotonous diets resulting in some animals expressing abnormal and stereotyped behaviors, considered an indicator of poor welfare (20).

## Forage Diversity and Generalization to Other Contexts

As expected from an evolutionary point of view, the ability of animals to compensate for the variability imposed by their environment will be a function of the individuals' phenotypic plasticity (50). Such plasticity may also be interpreted as behavioral flexibility, allowing for a rapid pathway for adjusting to environmental changes that exceeds the rate of evolutionary genetic change (51). Behavioral flexibility may be acquired when animals become familiar with solving foraging problems, generalizing their problem-solving abilities to other contexts, and situations imposed by a changing environment. There is evidence for this process to occur under natural and artificial settings. For instance, models with hummingbirds suggest that environmental heterogeneity (e.g., changes in temperature, water and food availability) are linked to problem-solving abilities, innovation, and exploration that allow individuals to better adapt to the unpredictable conditions of their environment (52). Environmental enrichment programs that allow farm animals show a more flexible foraging behavior lead to reductions in chronic stress due to confinement (53). Sheep have been found to predict and form expectations about the amounts of food that they are receiving, and to control an aversive event in order to access food, showing problem-solving abilities that allow for adjustments to new situations stemming from challenges experienced and solutions achieved in previous tasks (46). This plasticity acquired by the appraisal of novel situations relative to the individual's abilities and past experiences suggests that animals challenged by less predictable environments may be more likely to show a broader range of coping strategies in response to changing environmental conditions than animals living in more stable and predictable conditions (54). Behavioral flexibility may be also influenced by early life conditions as individually housed calves had learning deficits relative to calves housed in a dynamic group with access to their mothers (55).

## Variable Landscapes and Eustress

Access to pasture for animals kept in confinement provides some health-related welfare benefits to cows, even when diets in confinement are nutritionally balanced and cover all of the animals' physiological needs (health domain in the Five Domains Model). For example, at least in some circumstances, cows on pasture have a lower incidence of lameness (56) and mastitis (57) than cows kept indoors. If given the choice, cows will spend a significant proportion of time on pasture, mainly at night (58). Moreover, by using operant responses to assess motivation, it has been shown that cows value access to pasture as highly as fresh feed (59). It might be suggested that access to pasture provides an opportunity to experience a more diverse, stimulus-rich environment than indoor housing. Although boredom in animals has received little empirical study, research done in several species suggests that monotonous environments caused an increased motivation for diverse stimuli, consistent with the hypothesis that animals kept in barren environments may experience boredom or something like it (60). Cattle in pens with *ad libitum* access to a monotonous forage displayed contrafreeloading, spending energy (they pushed a gate) to obtain a forage which was simultaneously available in a feeder and in abundance (61). This behavior could be interpreted as a form of environmental enrichment, given that the housing environment was barren and the animals had limited social contact (61). Alternatively, pushing a gate may have been perceived as rewarding if this behavior attenuated boredom, created a sense of control over the environment or allowed the animals to experience “a sense of victory” by handling a doable challenge (61).

Consistent with the research described for cows in confinement, and for zoo animals exposed to a choice between different environments (27, 28), the welfare of ruminants grazing in extensive systems may benefit from the opportunity to choose across different locations in the landscape. Nevertheless, preference in dominant animals within a social group may overcome spatial preferences by subordinate animals, which may lead to frustration. Food preferences and social interactions both influence choice of foraging location by sheep (62), although when animals experienced toxicosis after eating certain foods, dietary preferences overrode social influences (62).

Access to locations that require complex tasks like moving across rugged terrain or uphill may represent challenges that elicit a higher degree of behavioral diversity in grazing animals (environmental domain in the Five Domains Model). Foraging enrichments in confinement are in general designed to facilitate the physical expression of feeding behaviors such as food-searching and food consumption, but not to facilitate complex tasks related to food acquisition (30), although recent research on cognitive enrichment shows positive effects on animal welfare (63). We propose that the equivalent of complex task-solving processes for animals grazing in extensive systems entails building a diet from a diverse and complex landscape with different biotic (e.g., plants with diverse chemistries and structures) and abiotic (e.g., slope, rough terrain, rocky outcrops) challenges. Foraging across different spatial scales, from regions and landscapes to plant communities and patches could be viewed a “natural cognitive enrichment program” that enhances animal welfare by providing a form of enrichment, creating a sense of control over the environment, or allowing for the realization of a task that leads to a positive emotional state. In contrast, lack of biotic and/or abiotic challenges, similar to those observed in barren environments lead to boredom, frustration, helplessness, and depression (5). Supplying grazing animals with the opportunity to interact with a more sophisticated environment by challenging their cognitive abilities with chances to gain environmental control or to anticipate rewards represents an appealing approach to enhance their welfare, supported by the positive results observed for animals living in captivity (64). These conditions at the spatial scale may facilitate the acquisition of positive emotional states, induced by a successful coping with a complex cognitive challenge rewarded by the formation of a balanced diet. Consistent with this idea, complex behavioral tasks rewarded by food improve the welfare of intensively housed pigs by providing adequate cognitive challenges that generated successful coping and positive emotional states (63, 65). In addition, structural (climbing racks) and cognitive enrichment (drinking water as a reward for a correct choice) improved different aspects of behavioral competence (e.g., visual four-choice discrimination tasks and reactions to external challenges) in goats exposed to stressful situations relative to animals exposed to barren environments with easy access to water (64). It has been hypothesized cows remain longer at feeding sites in rugged heterogeneous pastures, with more diverse vegetation and nutrient profiles, than in homogeneous pastures as variability in biotic and abiotic factors reduce satiety and increase residence time at the more complex feeding sites (66). In contrast, monotonous landscapes of uniform topography promote satiety and reduce the time spent at individual feeding sites (67). Cows born and raised under the environmental challenges of the Chihuahuan Desert were farther from water and spent less time at water than naïve cows of the same breed grazing at the same location, but born and raised in a humid environment with gentle topography and lush vegetation (68). During winter and early summer (drought conditions), naïve cows selected diets with lower crude protein content than cows born and raised in the desert (68). No welfare parameters were measured in this study, but it is likely that “desert cows” experienced a sustained cognitive enrichment by the association of successful coping with a demanding behavioral task (i.e., moving in rugged terrain, uphill and away from water in a dry environment) rewarded by food. Interestingly, cows born and raised in the desert, moved to lush pastures for 3 years and then returned to the desert, displayed behavioral patterns similar to cows that spent their whole life at the desert (68).

## Variable Landscapes and the Thermal Environment

Trees, shrubs, or long grass, as well as abiotic factors such as topography also provide a diversity of structural arrangements in the landscape that contribute to reduce the incidence of thermal stress in animals living under natural conditions. Thermal stress is a direct welfare problem, as it causes discomfort and it can significantly reduce access to pasture in grazing animals (58). Use of shade is likely to be the most feasible strategy for grazing ruminants. Depending on the quality of shade, provision of shade will reduce radiant heat load by 30–70% (69). Even in temperate climates, provision of shade has positive effects on heat load and production in grazing ruminants (70). The thermal environment plays an important role in determining livestock distribution (71), and thus factors in the landscape such as aspect, slope, type of terrain, type of vegetation also provide “tools” that allow animals gain environmental control or anticipate rewards (i.e., approach to their thermoneutral zone). Such tools are absent in flat terrains without shelter. On sunny summer days, cows have been observed spending considerable time (8 h) under shade trees near water (72). Contrastingly, during winter cattle exhibited heat seeking strategies of grazing south slopes during the day and not resting under shade trees and laying down at night on warmer ridges (72). On cold days cattle would move to lower, sheltered areas that were warmer (72), and on windy days cattle rest in sinkholes sheltered from the wind, possibly creating a more thermally neutral microclimate (71). Effective shelter during cold weather may also entail dry grass or shrubs when sheep graze in areas with such structural diversity in the vegetation, leading to substantial improvements in lamb survival under cold stress (73). Thus, biotic and abiotic factors that lead to successful coping such as efficient thermoregulation could be viewed as “natural” enrichment elements with potential to enhance animal welfare.

## Rangeland Diversity, Management, and Preparedness to the Unknown

Social and psychological research has placed emphasis in recent years on positive outcomes to stress-related experiences that breed resilience in organisms (74), instead of negative emotions and chronic stress that promote illness. A similar approach could be followed in natural systems by maintaining or enhancing their chemical and structural diversity, which in addition to services like improvements in the efficiency of resource capture, nutrient cycling and stability (75, 76), may promote improvements in animal welfare. Providing new chemicals to the landscape like medicinal PSC (e.g., with the introduction of herbs, shrubs or trees) will benefit the nutrition and health of grazing animals (77, 78), thus addressing the nutrition and health dimensions of the Five Domain Model. The benefits of plant diversity on animal welfare may also need to be pondered in relation to the nature of the assemblage of plant species presented to herbivores. For instance, when subjects are offered a choice of foods under experimental settings to understand their specific nutrient requirements through their diet-building abilities [e.g., the geometric framework of diet selection; (79)], the foods presented are unbalanced but designed in such a way that allows for the construction of a mixed balanced diet that meets the specific nutrient requirements of the individual. Extrapolating from this controlled setting to natural systems, the foods presented in the plant community should be such that different individuals under different physiological states should be able to build a balanced diet. If mixing diverse foods does not lead to meeting specific nutrient and medicinal needs, animals may experience negative emotions such as frustration in response to suboptimal diets, in addition to the direct negative impacts of unbalanced diets on fitness. Thus, it is important to consider the herbivores' foraging preferences when managing grazing environments for improvements in animal welfare and productivity. As an example, it has been shown that beef heifers grazing in natural grasslands use low-quality tussocks in order to harvest strategic amounts of dry matter during the day, given that the high productivity of these plants offer large intake rates (80). Intake of tussocks is then complemented with the consumption of high-quality herbs and grasses (of lower productivity) present in inter-tussock areas (80). Originally, farmers tended to eliminate low-quality tussocks, regardless of their abundance in the plant community, as they were considered problematic for animal production. After exploring the animals' feeding preferences, tussocks are now considered beneficial when present in the plant community below a certain frequency threshold such that animals build a diverse diet, where tussocks optimize the amount of biomass harvested daily and herbs and grasses in the inter-tussock areas provide the nutrients needed to optimize the digestibility of tussocks in the rumen (81). Managers also offer proportions of grass and clover in the landscape that match the grazers' preference for these forages in order to foster an optimal use of food resources by herbivores (82); such arrangement may also contribute to animal welfare improvements as it offers the conditions needed to successfully complete the task of building a balanced diet. Thus, fostering the “right” diversity, i.e., arrays of complementary plants that when mixed lead to the realization of a balanced diet is essential.

Appropriate challenge is a key concept in environmental enrichment, referring to the problem that is potentially solvable through the application of an animal's cognitive and behavioral skills (30). Under this scenario, managers may be able to “enrich” a certain landscape by providing the supplements or plant species that complement the chemical composition of the existing vegetation such that animals are able to build “optimal diets” that provide an appropriate mix of nutrients and medicinal PSC. Environments of low nutritional quality reduce the fitness and welfare of grazers and browsers (13), but the same is true when plants are high in nutrients (38). Non-complementary plant species that provide excesses of nutrients (e.g., crude protein) lead to stress and food aversions because excessive or frequent ingestion of such foods produce high levels of byproducts of fermentation that are toxic [e.g., ammonia or acid loads; (38, 83)].

Animals faced with the problem of building a diet from an array of diverse and complementary alternatives may be more adapted to maintain their fitness and welfare in response to future challenges triggered by shifts in vegetation like the predicted reductions in crude protein content in grasses in response to increased ambient temperatures (15). It has been proposed that as climatic warming reduces grass protein concentrations, woody species increase in abundance and grassland habitats decline for growing populations of herbivores, wild, and domestic species may need to compensate by relying less on grass and more on browse, which contains greater concentration of PSC (15, 84). In addition, the pattern of protein reduction in grasses may also increase the reliance on protein-richer eudicots, with greater potential for toxicity due to the greater concentration of PSC in these flowering plants (85). Plant secondary toxicity may also be exacerbated with the predicted increases in ambient temperature because toxins interfere with thermoregulation (86, 87). Detoxification pathways are thermogenic and toxins uncouple mitochondrial oxidative phosphorylation, which also generates heat (88). The imbalanced nature of herbivore diets and the presence of toxins, which affect the thermal balance, may increase the likelihood of heat stress at high ambient temperatures (88). These emerging problems may require building diets of lower PSC content in landscapes where the concentration of protein is declining. Finding appropriate locations in the landscape to dissipate heat under warmer conditions and the challenge of temperature-dependent toxicity will be more relevant for future generations of herbivores. Adaptability in these predicted scenarios is expected to be greater for animals previously exposed to solving the problem of building balanced diets from complex arrays of unbalanced alternatives and complex landscapes with a diversity of biotic and abiotic factors that foster cognitive enrichment. Trans-generational diet-building abilities of offspring as observed in cattle (89) and sheep (90) may also contribute to more efficient adaptations to future environmental challenges.

## Conclusions

We submit that chemical and structural diversity breed animal resiliency and adaptability to current and future challenges imposed by the inherent dynamic conditions of rangelands. Positive outcomes to stress-related experiences may enhance behavioral competence and lead to positive emotions that benefit animal welfare. Using this concept, managers should promote resilience and plasticity in animals by enhancing chemical and structural diversity in rangelands (i.e., through targeted grazing treatments, revegetation efforts that increase plant species diversity, or by strategic distribution of water points that enhance animal distribution in the landscape), thus creating “natural cognitive enrichment programs” that enhance animal welfare and better prepare animals for future challenges inherent of living in these dynamic and variable landscapes.

## Author Contributions

JV drafted the manuscript. JV and XM wrote it in collaboration and read and approved the final manuscript.

### Conflict of Interest Statement

The authors declare that the research was conducted in the absence of any commercial or financial relationships that could be construed as a potential conflict of interest.
